# Exploring the Relationship between Fusion Genes and MicroRNAs in Cancer

**DOI:** 10.3390/cells12202467

**Published:** 2023-10-17

**Authors:** Saurav Panicker, Gautham Chengizkhan, Ravi Gor, Ilangovan Ramachandran, Satish Ramalingam

**Affiliations:** 1 Department of Genetic Engineering, School of Bio-Engineering, SRM Institute of Science and Technology, Kattankulathur, Chengalpattu 603203, Tamil Nadu, India; sauravgenetics23@gmail.com (S.P.); ravigor95@gmail.com (R.G.); 2 Department of Endocrinology, Dr. ALM PG Institute of Basic Medical Sciences, University of Madras, Taramani Campus, Chennai 600113, Tamil Nadu, India; gauthamc@outlook.com

**Keywords:** fusion genes, microRNAs (miRNAs), introns, precision therapy, aberrations, post-transcriptional regulation

## Abstract

Fusion genes are key cancer driver genes that can be used as potential drug targets in precision therapies, and they can also serve as accurate diagnostic and prognostic biomarkers. The fusion genes can cause microRNA (miRNA/miR) aberrations in many types of cancer. Nevertheless, whether fusion genes incite miRNA aberrations as one of their many critical oncogenic functionalities for driving carcinogenesis needs further investigation. Recent discoveries of miRNA genes that are present within the regions of genomic rearrangements that initiate fusion gene-based intronic miRNA dysregulation have brought the fusion genes into the limelight and revealed their unexplored potential in the field of cancer biology. Fusion gene-based ‘promoter-switch’ event aberrantly activate the miRNA-related upstream regulatory signals, while fusion-based coding region alterations disrupt the original miRNA coding loci. Fusion genes can potentially regulate the miRNA aberrations regardless of the protein-coding capability of the resultant fusion transcript. Studies on out-of-frame fusion and nonrecurrent fusion genes that cause miRNA dysregulation have attracted the attention of researchers on fusion genes from an oncological perspective and therefore could have potential implications in cancer therapies. This review will provide insights into the role of fusion genes and miRNAs, and their possible interrelationships in cancer.

## 1. Introduction

Fusion genes are important drivers of cancer, and they are implicated in the initiation and progression of several cancers. MicroRNAs (miRNAs/miRs) play a key role in the post-transcriptional regulation of many genes, and therefore, miRNA dysregulation could lead to the development of various cancers. In the quest to explore the clinical application of fusion genes and to enhance the outcome of patients in clinical settings [1,2], it is imperative to understand the interplay between fusion genes and miRNAs. It is essential to improve and create precision drug targets against various cancers in this present age of tailor-made medicine [1]. Surprisingly, the dynamics between fusion transcripts and miRNAs during carcinogenesis remain vastly underexplored. The myriad signalling mechanisms employed by the fusion genes in driving the cancer have been studied but their impact on miRNAs remain obscure. In our recent review, we delineated how fusion genes regulate the cancer stem cells (CSCs) and further we discussed how the fusion proteins can serve as ideal targets for future therapies to specifically eliminate the CSCs without affecting the healthy normal cells and stem cells [2]. Such fusion protein-based targeted-oncotherapies to eradicate CSCs can be highly efficacious in preventing tumor relapse with minimal side effects [2] and can eventually achieve the essential objectives of precision medicine [3]. In the present review, we explored the interactions between fusion genes and miRNAs, which may provide more insights in understanding the mechanisms involved in the initiation and development of tumors at the transcriptome level. It is noteworthy to remember that the significance of fusion genes on miRNAs has predominantly been neglected. Nevertheless, fusion events have been related to miRNA aberrations [4]. In this review, we have explored how the fusion genes affect miRNA expressions and vice-versa in cancers.

**Figure 1:**
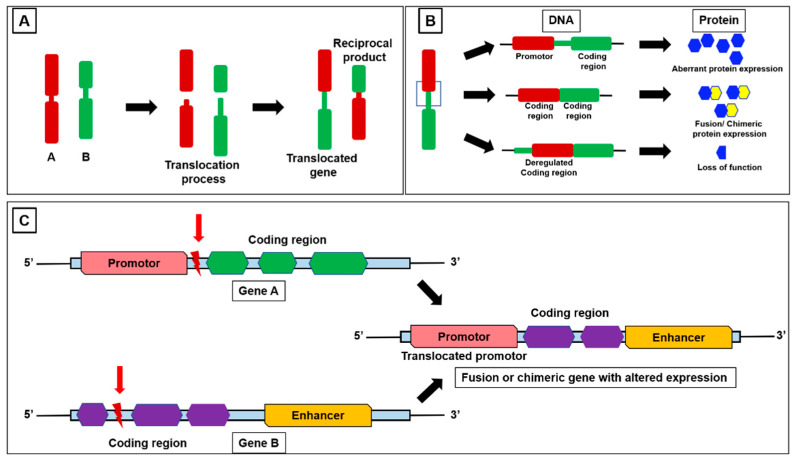
Schematic representation of (**A**) fusion gene translocation, (**B**) fusion or chimeric protein production via rearrangement of promoter and coding region and (**C**) fusion or chimeric gene production via rearrangement of promoter and coding region between genes.

The human genome provides all the information that an organism requires to function. Genomic rearrangement (GR) also known as chromosomal aberration (CA) that occurs inside the cells can result in various diseases including cancer. GR includes gene mutations, inversions, deletions, translocations, and copy number alterations (CNA) of a single nucleotide of DNA or a piece of DNA. A recent study shows an association between structural variations (SV) and gene expression in cancer, demonstrating that SV breakpoints near genes can impact the gene expression independently of CNA. These findings help us to understand the molecular mechanisms underlying the cancer development and progression [5]. Fusion genes are formed due to GR (Figure 1), although not all the GR changes at the genetic of transcript level may result in functional nucleic acids or fusion-protein [6]. These rearrangements can occur in specific regions where unknown genes are present. A list of fusion genes and translocation events that cause aberrant changes in miRNAs is provided in Table 1.

Several fusion genes are reported to contribute to carcinogenesis in various cancer types. In the year 1960, Breakpoint cluster region-Abelson murine leukemia viral oncogene homolog 1 (*BCR-ABL1*) was the first fusion gene identified using cytogenetic analysis [26]. Advancement in the DNA sequencing technologies has led to the identification of more fusion genes in many different cancer types [27]. Fusion genes namely *BCR-ABL1*, Paired box gene 3—Forkhead box protein O1 (PAX3-FOXO1), Fibroblast growth factor 3—Transforming acidic coiled-coil containing protein 3 (*FGFR3-TACC3*), Splicing factor proline and glutamine rich–Transcription factor binding to IGHM enhancer 3 (*SFPQ-TFE3*), Transmembrane protease, serine 2—ETS-related gene (*TMPRSS2-ERG*), Echinoderm microtubule-associated protein-like 4—Anaplastic lymphoma receptor tyrosine kinase (*EML4-ALK*) and MYB–Nuclear factor 1 B-type (*MYB-NFIB*) were shown to cause various types of cancer [27]. The *PAX3-FOXO1* fusion gene is a result of the chromosome 2 and chromosome 13 reciprocal translocation and is specific to the alveolar rhabdomyosarcoma (aRMS) [28]. One of the most common fusion genes in several cancers namely *FGFR3-TACC3* is reported in bladder, brain, and lung cancers. In bladder cancer, the *FGFR3-TACC3* fusions *TACC3* is a common partner that plays an important role in stabilizing and organization of the mitotic spindle to allow proper chromosome segregation [27,29]. In brain cancer, the *FGFR3-TACC3* fusion was caused by the tandem duplication on 4p16.3, which led to the loss of the 3′-untranslated region (3′-UTR). Loss of 3′-UTR resulted in the loss of miR-99a gene regulation and resulted in enhancement of the fusion gene expression and promotes tumorigenesis in glioblastoma [30]. The *EGFR3-TACC3* gene fusion in nonsmall cell lung cancer confers resistance to all the generation of the EGFR tyrosine kinase inhibitors (TKIs) [31]. The *SFPQ-TFE3* gene is found in 1.2% of the clear cell renal cell carcinoma patient samples. The integrative pathway analysis has showed the importance of the CHL/HIF, PI3K/AKT pathway, chromatin remodeling/histone methylation pathway [29]. The *TMPRSS2-ERG* is the most common fusion gene found in almost 50–70% of prostate cancer patients. This results from the fusion of the androgen-regulatory promoter of the *TMPRSS2* gene with ERG, thereby ensuing ERG overexpression. This promotes cancer progression and metastasis in prostate cancer [32]. The inversion joining of the gene EML4 and ALK in chromosome 2 has resulted in the *EMLK-ALK* fusion gene. It is found in approximately 4% of the nonsmall cell lung cancer (NSCLC), and it shows oncogenic effects by overexpressing ALK tyrosine kinase. This results in constitutive expression of the downstream signaling cascade, such as AKT, MAPK, and STAT3 [33,34]. *MYB-NFIB* fusion gene is formed by deleting the 3′-UTR regions of the MYB mRNA. This has allowed the MYB-NFIB fusion mRNA to be undetected by the regulatory miRNA.

## 2. MiRNA in Precision Medicine and Precision Oncology

Precision medicine has emerged as a modern cornerstone in formulating personalized cancer therapies for each patient [35,36] based on the tumor’s specific molecular attributes [36,37]. Precision oncology is the molecular profiling of tumors [35] that delves more into the genomic constitution of the tumor. It is more convenient and effective in selecting more therapeutically efficacious treatment regimens specific to the tumor’s unique characteristic profile of an individual patient [1,37]. Therefore, it is a prerequisite for us to understand the role of precision oncology and medicine in formulating personalized therapies for improving the patient outcomes [37]. Integrating transcriptomics data into the precision oncology paradigm aids in circumventing the inefficiency of genomic data in accurately deciding treatment regimens and predicting treatment outcomes [38]. MiRNA transcriptomics in cancer has been assessed in the past to understand correlations between miRNA expression patterns and tumor clinicopathological features [39]. It is crucial to investigate the mechanisms by which the fusion genes modify the miRNA transcriptome, which may provide interesting and more valuable insights into the future of oncobiology [4,40,41,42]. Combining the fusion gene expression profiles with miRNA expression profiles can shed more light on their mechanisms and help us to delineate the tumor’s molecular portrait. Hence, it is essential to study the miRNA aberrations caused by driver fusion oncogenes during carcinogenesis in detail, which will help to render more precise clinical recommendations.

## 3. Fusion Genes and Chimeric Fusion Transcripts (Fusion RNAs)

Fusion genes and/or fusion transcripts (fusion RNAs) have been the critical oncogenic drivers in a myriad of cancers [3,43,44]. Fusion genes are aberrantly activated because of genomic instability [3,44]. It is clear from the previous studies that the rearrangement of fusion genes can be activated at both the DNA and RNA levels [45]. At the DNA level, chromosomal rearrangements like inversions, deletions, amplifications, translocations, and chromothripsis can give rise to fusion genes that, when transcribed, produce more fusion transcripts and subsequent production of fusion proteins after post-translational modifications [3,43]. Also, it is imperative to understand that both intra-chromosomal and inter-chromosomal rearrangements can activate the fusion genes. At the RNA level, the abnormal functioning of the splicing machinery can result in fusion RNAs that drive tumorigenesis [3]. Other genomic events, such as trans-splicing [45], cis-splicing [3], transcriptional readthrough errors [45,46], and intergenic splicing [47], generate fusion RNAs that play key roles in cancer initiation and progression. The latest discovery in the area of fusion RNAs represents a new class of fusion RNAs called cross-strand chimeric RNA (cscRNA) that may have beneficial implications in cancer diagnosis and treatment [44].

Cancer-specific fusion oncogenes are specifically expressed in cancer cells but not in normal cells [2]. Fusion genes are true genetic drivers of oncogenesis [38,47] that are functionally responsible for driving the initiation and progression of many tumors [48,49]. In a few cancers, the fusion gene will be the only driver of mutation for that particular cancer [49]. Fusion genes are cancer-specific etiological aberrations correlated to the tumor phenotype [48,50,51]. Since these genes have diverse features and characteristics, they are typically used as drug targets for precision oncotherapies [2,38], however, it is unequivocal that not all fusion genes contribute to carcinogenesis [3], as these genes also play a role in normal physiological functions [52].

## 4. Fusion Genes and MiRNAs

### 4.1. MiRNAs: Key Players in Gene Expression and Tumorigenesis

**Figure 2:**
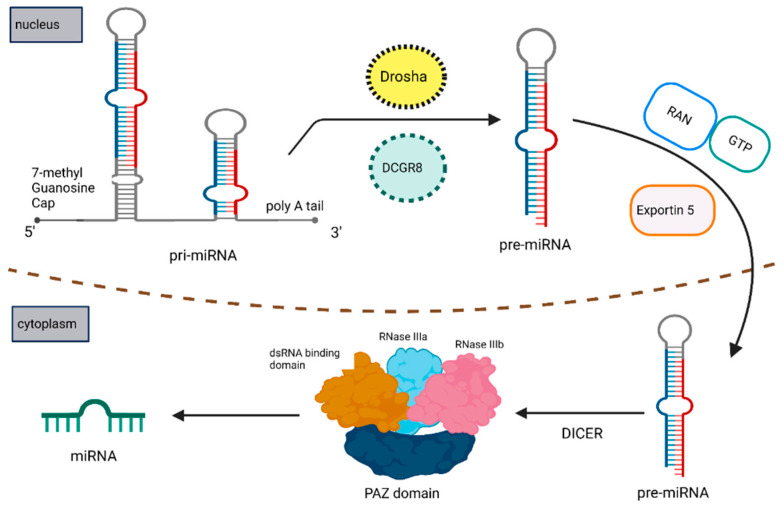
Schematic representation of miRNA biosynthesis and its maturation.

**Figure 3:**
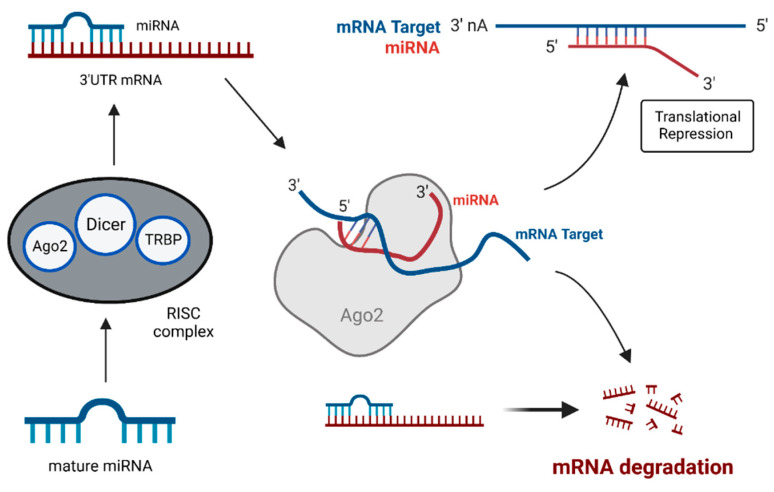
Schematic representation of the role of miRNAs in miRNA-mediated translational inhibition and mRNA destabilization.

MiRNAs are single-stranded short noncoding RNAs of 18–22 nucleotides in length that lack open reading frames (ORF) and do not code for any proteins [50,51,52,53,54]. MiRNAs are the master regulators of eukaryotic gene expression, as they are involved in regulating the gene expression. They bind to the 3′-UTR (untranslated region) of their target mRNAs to bring about miRNA-mediated translational repression and/or miRNA-induced mRNA degradation (Figure 2 and Figure 3) [51,54,55]. MiRNA dysregulation caused by fusion genes disrupts the equilibrium of the transcriptional network in cancer cells and often initiates anomalous cell survival and cell death mechanisms [14,53]. Chromosomal translocations at the miRNA loci play an important role in the deregulation of miRNA and induce the tumor initiation and progression [14,54,55,56].

### 4.2. Mechanism of Fusion Gene-Mediated Aberrant miRNA Expression

Fusion genes contain intronic regions coding for miRNAs. Most of the parent genes that undergo translocations to produce fusion genes consist of many intronic regions that code for miRNAs [41]. The first step involves the exchange of coding segments between two different genes [14,56]. This is the most frequently observed translocation process, often leading to a disruption of the gene structure coding for the miRNA. The subsequent miRNA deregulation due to the disrupted gene structure is more common in human myeloid malignancies and soft tissue tumors [14]. In contrast to the above types of translocations, which normally lead to the activation or overexpression of certain miRNAs, there exists another class of translocations. This class does not directly involve the miRNA transcript-coding region in the fusion, yet it leads to miRNA dysregulation and promotes tumorigenesis [4,57]. Out-of-frame translocations that occur in the regions upstream to the miRNA-encoding introns can further lead to the deregulation of intronic miRNAs [4,41]. Such translocations that occur only at the upstream regulatory regions can also alter the expression pattern of the downstream intronic-miRNA coding region, however, these intronic regions do not directly take part in the fusion process [4,41]. Classical fusion that causes miRNA dysregulation involves the region that codes for that particular miRNA to be the crucial factor of the breakpoint cluster. In all the above-mentioned cases, the miRNA sequence remains undisturbed, but its expression gets altered as a result of the translocations at the promoter regions [4,41]. Fusion genes formed via such patterns will have repeated inclusion of the same miRNA sequence but with a different 5′ upstream sequence [4,41]. In these cases, multiple chromosomal segments undergo fusion at the promoter of a miRNA-coding gene, thus forming a convergent fusion group that modulates the transcriptional expression of a single common miRNA [14,41]. Such promoter-switching events result in the aberrant activation of miRNA that can cause an imbalance in the equilibrium of the transcriptional network in a cell, eventually leading to tumor initiation [4,14,41]. These types of miRNA deregulations are more prevalent in B- and T-cell lymphomas [14].

The complexity of chromosomal translocations includes translocation of the promoter/enhancer elements of miRNA genes upstream to the protein-coding region of another gene [14,41], promoter elements of one miRNA exchanging with the promoter elements of another miRNA [14,41], miRNA coding region fusing with another miRNA coding region to give rise to a new miRNA cluster [14,41], two genes undergoing fusion that can cause disruption of intermittent introns coding for miRNAs [4,41], and miRNA loci fusing downstream to the upstream regulatory elements of a protein coding gene. These are the most common intricacies that can cause dysregulation in miRNA expressions, and subsequently lead to tumor development.

### 4.3. Fusion-Induced Promoter Transposition

**Figure 4:**
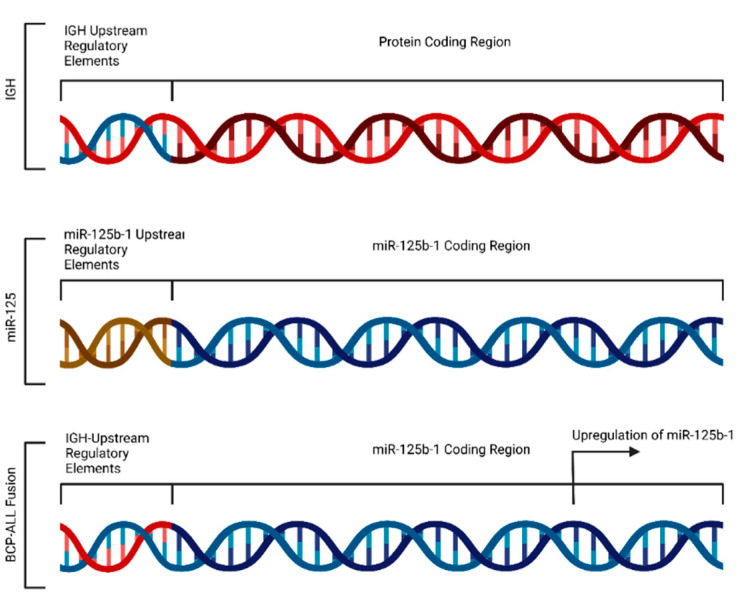
Schematic representation of upregulation of miR-125b-1 in *BCP-ALL* caused by a fusion event.

As discussed earlier, the chromosomal translocation that triggers tumorigenesis can lead to the activation of specific miRNAs that significantly contribute to tumor progression [7,41]. A frequent translocation found in myeloid cancers is t(2;11)(p21;q24), which activates the miR-125b-1 on chromosome 11 [7]. Upregulation of miR-125b-1 due to chromosomal translocation accelerates the rate of cellular transformation in myeloid tumors [7,58]. B-cell precursor acute lymphoblastic leukemia (*BCP-ALL*) is often caused by the chromosomal rearrangement at t(11;14)(q24;q32). The translocation of 11q24 to the immunoglobulin heavy chain (IGH) locus 14q32 transactivates the critical region that primarily encodes for miR-125b-1 [7]. The loci that code for miR-125b-1 are juxtaposed downstream to the upstream regulatory regions of the IGH gene locus, ensuing miR-125b-1 upregulation in *BCP-ALL* (Figure 4) [58]. Upregulation of miR- 125b-1 in *BCP-ALL* is a classic example that explains the alterations in miRNA expression due to fusion-induced dispositions of miR loci under the modulation of different regulatory regions. This specific miR-125b-1 overexpression promotes the oncogenic transformation of B-cell precursors in *BCP-ALL* [41,56].

### 4.4. Altered Expression of miR-29 in Lymphomas and Sarcomas

Recurring instances of translocations at 7q32.3 have been consistently observed in ALK-negative large cell lymphomas (ALCL), culminating in the upregulation of miR-29 [8]. miR-29 holds a pivotal role in the pathogenesis of cancer. Its influence spans diverse oncogenic processes encompassing epigenetics, proteostasis, metabolism, proliferation, apoptosis, metastasis, fibrosis, angiogenesis, and immunomodulation. Despite its primary characterization as a tumor suppressor, there exists a disparity within the literature concerning its potential as an oncogene. Notably, miR-29’s role in cancer is intricately contingent upon contextual factors, necessitating further investigation for comprehensive elucidation. The miR-29 governs diverse cellular functions, including apoptosis, proliferation, and angiogenesis, in cancer cells [59]. In the context of breast cancer, divergent outcomes have been documented in studies concerning miR-29’s role. While some studies propose its participation as a tumor promoter, others underline its tumor-suppressive attributes. The functional ramifications of miR-29 on colony formation and tumor growth in breast cancer cell lines vary considerably across investigations. Conversely, in pancreatic cancer, the prevailing consensus from multiple studies points to the tumor-suppressive function of miR-29. The introduction of miR-29 mimics into pancreatic cancer cells has demonstrated inhibition of tumor growth, migration, and invasion. Notwithstanding, another study has reported contrary effects, necessitating further scrutiny. In the realm of gastric cancer, miR-29 has emerged as a regulator of cell motility, counteracting the β-catenin pathway. This intrinsic regulation curtails the migratory potential of gastric cancer cells and impedes their metastatic capacity in vivo. The perturbation in miR-29 expression triggers an alteration in cancer cell invasion and migration by instigating various oncogenic pathways, including the Wnt/β-catenin pathway. Predominant evidence indicates miR-29’s tumor-suppressive role across assorted cancers. Over 85% of miR-29-associated cancer investigations underscore its inhibitory impact on diverse cancer-related targets. Nonetheless, the dualistic nature of miR-29 as both a tumor suppressor and an oncogene may pivot upon contextual nuances, necessitating further exploration to unravel its precise role within the cancer landscape [59]. Certain types of cancer and cancer subtypes are exclusively attributed to particular fusion genes. Within these tumor types, these specific fusion events play a prime role in initiating and propelling the entire process of carcinogenesis, regardless of the presence or absence of accompanying cooperating mutations. Indeed, the mere presence or absence of these fusion genes decisively dictates the neoplastic condition of the tissue in question. Remarkably, these cancer-specific fusion genes possess the capability to orchestrate tumorigenesis independently, even in the absence of additional cooperating mutations. Given that the genesis of these cancers is exclusively linked to specific fusion events intrinsic to the tumor tissue, these fusions have proven invaluable as highly sensitive markers for tumor detection and diagnostic purposes. Consequently, certain fusion genes fall under the classification of hallmark mutations that distinctly characterize these cancers. A prime illustration of this phenomenon can be observed in the case of *SYT-SSX* in synovial sarcoma, as well as *BCR-ABL* in chronic myeloid leukemia (CML) [60,61,62]. In alveolar rhabdomyosarcoma (ARMS), the t(2;13)(q35;q14) translocation generates the fusion protein PAX3-FOXO1. It has been shown that miR-29-a-3p is responsible for myofiber organization and myogenic structural protein expression. However, PAX-FOXO1 can also cause global alterations in miRNA expression, including miR-29-a-3p, which results in the aberration of this specific miRNA in myogenic pathways in ARMS [9].

### 4.5. Rearrangements of miR-15a and miR-16-1 Loci in Chronic Lymphocytic Leukemia (CLL)

**Figure 5:**
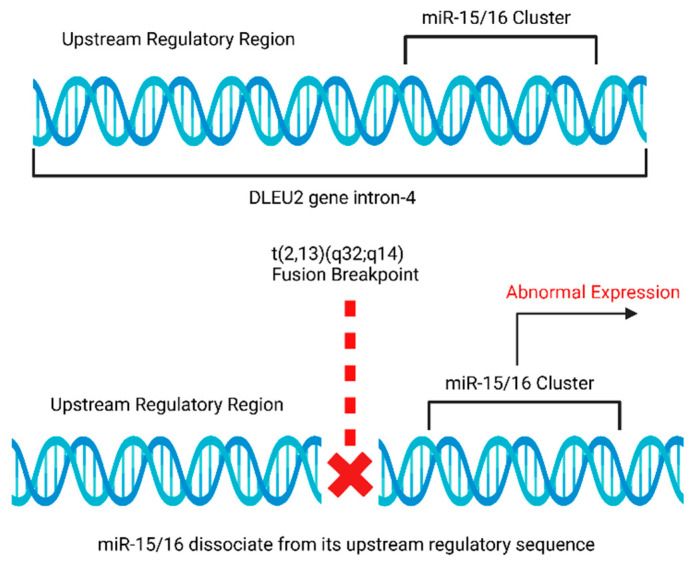
Schematic representation of disruption of the regulatory region due to translocation and alteration of the expression of miR-15a and miR-16-1.

The miRNA gene cluster, miR-15/16, involves two genes, miR-15a and miR-16-1, located within intron 4 of the *DLEU2* gene at the chromosomal band 13q14.2 [10,63]. The tumor suppressor function of miR-15/16 has been widely known and studied extensively [11]. It has been reported that miR-15/16 is frequently deleted in 13q14 deletion CLL cases, wherein 13q14 deletions are considered to be the major cause of cytogenetic aberrations in CLL cases [10,11,64], but it is either deleted or downregulated in most CLL cases [10]. miR-15/16 gene is located at or near the fusion breakpoint, and the t(2;13)(q32;q14) can alter the original upstream regulatory signals of miR-15/16 and thereby causing its altered expression (Figure 5) [10,11,63]. The downregulation of miR-15/16 contributes to stimulating leukemogenesis in chronic lymphocytic leukemia (CLL) [10,65,66,67]. Studies have shown that miR-15 and miR-16 can both repress *BCL2* post-transcriptionally to induce apoptosis [10,65]. However, the downregulation of miR-15 and miR-16 results in BCL2 overexpression that further favors the leukemic cells to evade apoptosis [65,68].

### 4.6. Translocation of miRNA-142 Causes c-MYC Overexpression in Acute Promyelocytic Leukemia (APL)

This specific region, 8q24.21, codes for *MYC* proto-oncogene, and the region, 17q22, codes for miR-142, translocation of the c-Myc locus, have been reported in leukemia patients affected by aggressive B-cell malignancy [12,13,14]. Further, the tumor cells with t(8;17)(q24;q22) translocation were shown to have a higher miR-142 expression [13]. It is evident that the t(8;17) fusion is presumably accountable for *MYC* activation because of promoter substitution [12,13]. It is clear from the above study that *MYC* is activated by the miR-142 promoter substitution as a consequence of the t (8;17) fusion event [12,13].

### 4.7. MiRNA Translocations in Mesenchymal Tumors and Benign Tumors

**Figure 6:**
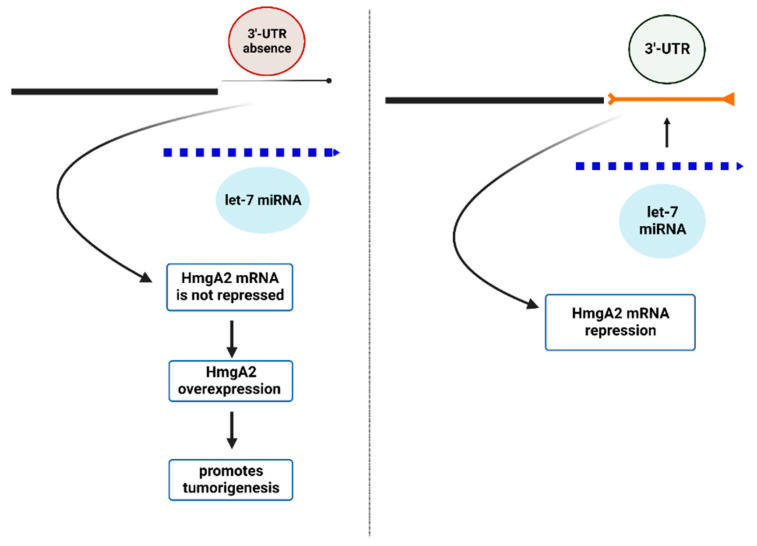
Schematic representation of chromosomal translocation at 12q15 and loss of miRNA binding site in the 3′UTR of mRNA as a classic example of a fusion event causing a disruption in the miRNA function by repressing the mRNA.

Chromosomal translocations at 12q15 lead to truncated HmgA2 mRNA, which results in the deletion of the complementary site for *let-7* miRNA binding. This impacts the deactivation of HmgA2 mRNA by *let-7* miRNA (Figure 6) [54,66,67]. The failed repression of HmgA2 mRNA promotes oncogenic transformation of cells and is considered to be a crucial characteristic feature of mesenchymal tumors and many benign tumors, including uterine leiomyomata [14,15,16].

### 4.8. BCL6 Juxtapositions to miR-28 Locus in Primary Central Nervous System Lymphoma (PCNSL)

**Figure 7:**
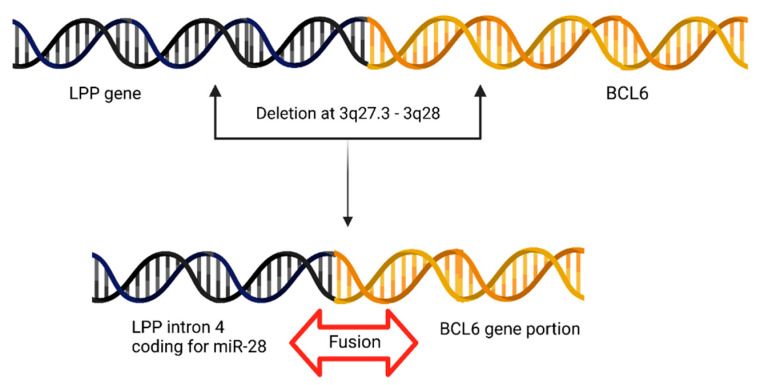
Schematic representation of the deletion at 3q27.3-3q28 causing miR-28 locus to be fused upstream to *BCL6* gene locus.

In PCNSLs, the fusion breakpoints in *BCL6* were studied. The deletions between 3q27.3–3q28 cause a fusion between *BCL6* and the *LPP* gene. This deletion juxtaposes *BCL6* in close proximity with a portion of the *LPP* gene (intron 4 of *LPP*) that presumably codes for miRNA-28. Hence, it is shown that deletion at 3q27.3 causes a fusion between miR-28 and *BCL6* (Figure 7) [17].

### 4.9. miR-17-92 Gene Cluster and Chronic Myeloid Leukemia (CML)

The reciprocal translocation at t(9;22) upregulates the miR-17-92 gene cluster in CML. Elevated expressions of the miR-17-92 cluster support the survival of *BCR-ABL*-positive cells [14,18]. Studies have shown that miR-17-92 polycistron is downregulated significantly after imatinib treatment in CML [14,18]. Additionally, the miR17-92 cluster was shown to regulate c-MYC, promoting tumor growth [19,69].

### 4.10. Fusion Genes Cause miRNA Overexpression in Solid Tumors

miRNA-21 is found to be significantly overexpressed in solid tumors [14,19]. The 17q23.2 region has been shown to code for miR-21 [14]. The miR-21 locus undergoes recurrent translocations and becomes upregulated in human glioblastoma [20,21,22]. Overexpression of miR-21 and miR-155 is considered to be the ‘oncogenic miRNA signature’ for major solid cancers, including colon, breast, lung, prostate and pancreatic tumors [19].

### 4.11. Abnormal miR-155 Levels in Burkitt’s Lymphoma Translocation

miRNA-155 plays a crucial role in the B-cell maturation process and B-cell functioning [70,71]. Overexpression of miR-155 results in immature B-cell malignancies [23,72]. Almost all B-cell lymphomas have miR-155 deregulation [12,13,15]. Burkitt’s lymphoma is a non-Hodgkin’s lymphoma affecting children caused by translocation at t(8;14)(q24;q32) [24]. Elevated levels of miR-155 were reported in children with Burkitt’s lymphoma [14,73]. Contrarily, there have been other reports stating that miR-155 upregulation is not consistent in all cases of Burkitt’s lymphoma [18].

### 4.12. AML1-ETO Fusion Protein Causes miR-223 Repression

AML1-ETO fusion protein expression is frequently observed in acute myeloid leukemia (AML) [25]. This fusion protein causes the silencing of miR-223 via the recruitment of HDAC and DNMT, which causes aberrations during the differentiation of myeloid progenitors, thereby promoting leukaemogenesis [25]. Thus, a fusion protein is involved in the epigenetic silencing of a miRNA to aid leukaemogenesis [25].

### 4.13. Clinical Relevance of Fusion Genes and miRNAs

Fusion gene detection remains of enormous significance in developing novel biomarkers for early diagnosis and developing a therapy [27]. Fusion genes cause genomic alterations that affect miRNA loci, ultimately leading to miRNA dysregulation [14,74]. Similar to fusion genes, miRNAs play an integral part in tumorigenesis as cancer-specific miRNA alterations have been discovered and such distinct “miRNA—fingerprints” aid in early cancer detection [75]. Further, the identification of miRNA-based markers and the development of tumor-specific miRNA expression profiles will be useful in the molecular subtyping of cancers and risk stratification [14,76]. The cDNA-based classifiers (EST-expressed sequence tags) are not promising in the grading/staging of poorly differentiated tumors, whereas cancer-specific miRNA- fingerprints are better in tumor classification and grading [77]. Some miRNA-coding genes are present on weak genome sites that are often susceptible to chromosomal translocations, thereby causing miRNA dysregulation via fusion events [78].

## 5. MiRNA-Mediated Cancer Therapeutics in Fusion Positive Tumors

**Figure 8:**
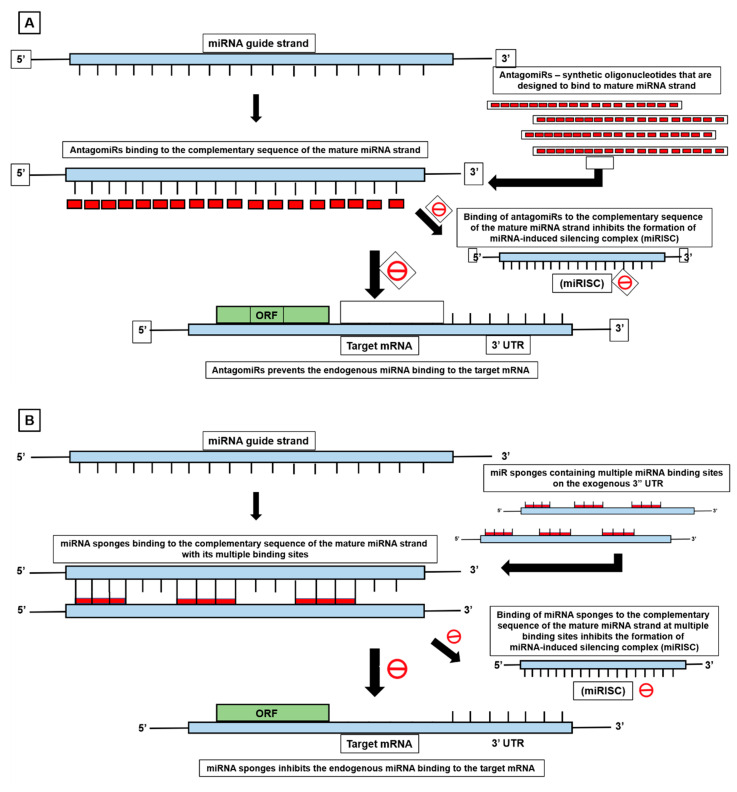

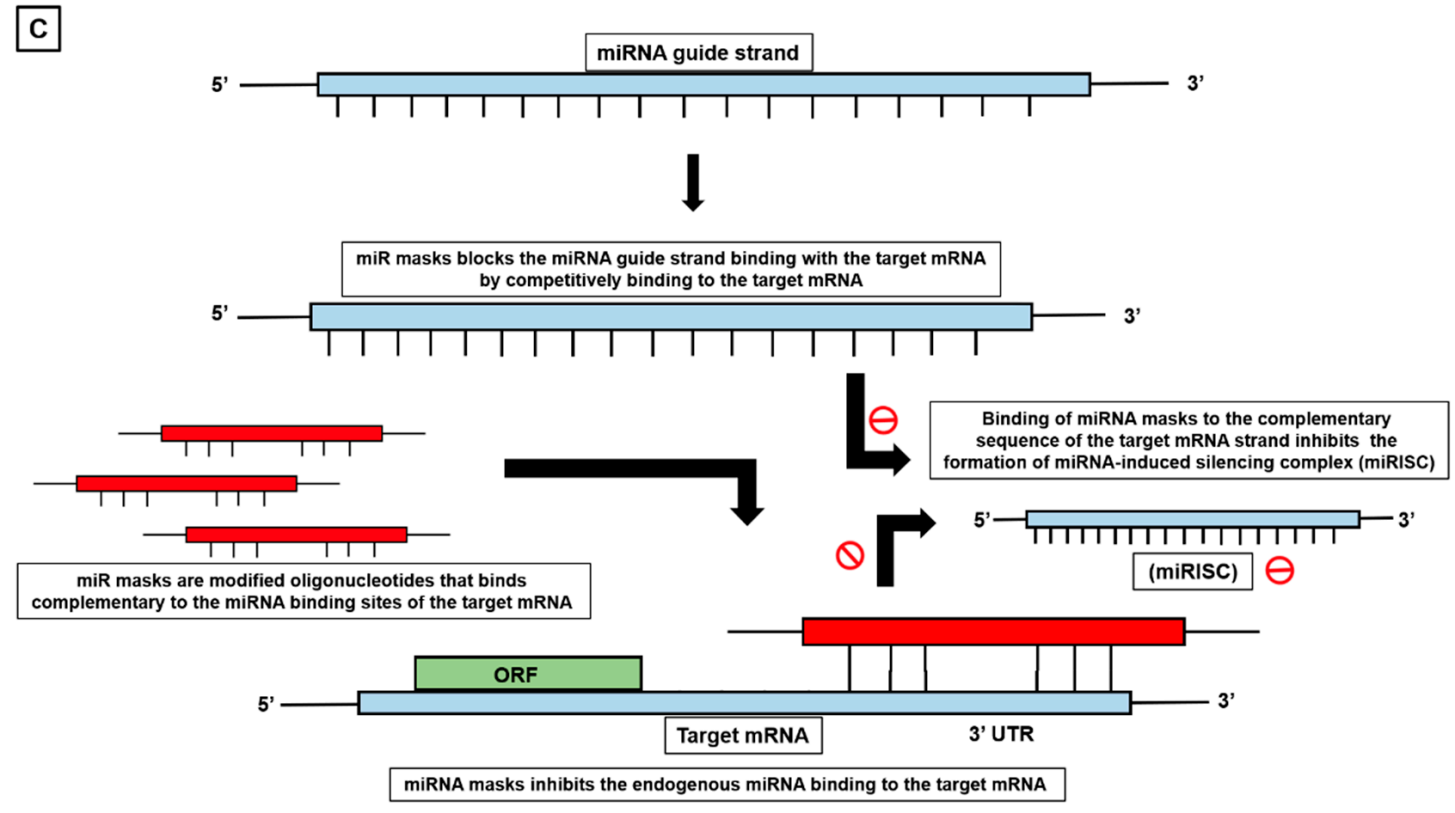
MiRNA-mediated cancer therapeutics (**A**) AntagomiR, (**B**) miR sponges, (**C**) miR masks.

MiRNA deregulation is strongly associated with the pathogenesis of numerous cancers [79]. Fusion-positive tumors are treated with kinase inhibitors (such as Gleevec) that block the activity of the fusion oncoprotein, which is responsible for cancer signal transduction [27]. Instead of using conventional methods to target fusion genes at the protein level, it is more effective to directly target the fusion-positive tumors at the transcriptome level. Some of the latest biotechnological advancements in miRNA-mediated cancer therapeutics are showing promising results. Some of the recently developed miRNA-based cancer therapeutics are discussed below. Antisense miRNA (anti-miR) oligonucleotides (AMOs) are developed and used to silence aberrant miRNA expression, which results in tumor growth inhibition [79,80]; however, AMOs have limitations such as nuclease resistance and irregular binding affinities. To overcome these limitations, AMOs are modified by making structural changes to the sugar backbone, such as 2′O-Methyl (2′OMe), 2′-Fluoro (2′F), 2′O-Metoxyethyl (2′MOE) and further that are chemically modified by adding phosphodiester bonds like phosphorothioate (PS) and N-mesyl- (μ-) and methoxyethyl-phosphoramidate [81]. Likewise, antagomiRs are short, specific oligonucleotides that are primarily designed to be complementary with the guide strand or mature miRNA strand. When the antagomiRs are transfected into the cells, they bind to the mature target miRNA strand by Watson-Crick base pairing. After binding to the guide strand, the antagomiRs interact with the desired miRNA and inhibits the formation of the miRNA-induced silencing complex (miRISC). This subsequently prevents the binding of the endogenous miRNA to its target mRNA, as seen in Figure 8A [82]. MiRNA sponge technology makes use of exogenous mRNA molecules that consist of repetitive miRNA binding sites to target specific miRNA [79]. Numerous miRNAs bind to the sponge RNA that acts as a sponge by trapping the miRNA (Figure 8B) [79]. The miR-Masks or BlockmiR (MicroRNA-Masking Antisense Oligonucleotides Technology) are miRNA blockers that prevent the miRNAs from binding to their target mRNA. MiRNA-masks are specifically gene-specific as they protect the mRNA from the aberrantly expressed miRNAs, as seen in Figure 8C [79]. The *BRD4-NUT* fusion gene drives a very rare carcinoma. NUT midline carcinoma (NMC) is a rare but lethal cancer. The introduction of miR-3140 in the NMC cell line has been shown to suppress *BRD4* expression by complementing and binding with the CDS region of the transcript. miR-3140 has been shown to reduce the BRD4-NUT protein in vitro and suppress tumor growth in vivo in a xenograft mouse model. This is one of the promising approaches to develop a miRNA-based medicine for cancer treatment [83]. Transfection of synthetic miRNAs can be used to upregulate the tumor suppressor miRNAs in cells to induce cell cycle arrest and apoptosis in cancer cells [81,84]. RNA zippers are also used to inhibit miRNA. It merges multiple copies of miRNA to form a duplex by joining them in an end-to-end manner, thereby disrupting the miRNA action. miRNA-based therapeutic approaches have lesser toxicity compared to conventional treatments [81]. Recent reports of miRNA-mediated translational activation that upregulates gene expression have opened a new avenue for scientists and clinicians to investigate if miRNAs can play a direct role in initiating the tumor and facilitating the cancer progression via upregulating the fusion genes [54].

## 6. Latest Update: ChimerDriver Exploits miRNAs in Detection and Classification of Oncogenic Fusion Genes

ChimerDriver (version python 3.6.12) is a recently developed software tool used to identify oncogenic fusion genes that drive mutations and distinguish them from nononcogenic fusion genes [85]. Fusion gene detection tools are completely based on machine learning techniques and deep-learning techniques [79,86,87]. However, most of the fusion gene detection software does not consider post-transcriptional regulations for predicting the oncogenic potential of a fusion gene. ChimerDriver software was recently designed and developed to overcome these limitations. It predicts the fusion genes and miRNA expression by integrating the information about miRNAs associated with the genes that are involved in fusions. ChimerDriver contains more than 300 miRNAs as its input features, and these miRNAs are added after exploring the possibility of targeting those genes that are involved in fusion genes. With the inclusion of miRNA features for assessing the oncogenic potential of fusion genes, it is reported that the number of false negatives decreased significantly and improved the recall value [79]. ChimerDriver software is shown to outperform both Oncofuse (version 1.0.7) and DEEPrior (version python 3.7) software, highlighting its prediction accuracy and oncogenic assessing capabilities [85,86,87]. The notable accomplishments and benefits of the newly evolved software further emphasize the importance of these detection tools in assessing the oncogenic potential of fusion genes and miRNAs [79].

## 7. Software and Databases to Identify miRNAs in Fusion Transcripts

Many open-source tools can be used to identify the fusion gene/transcripts from genome/RNA sequencing data, such as Spliced Transcripts Alignment to a Reference-Fusion (STAR-Fusion) (version 1.12.0), nFuse (version 0.2.1), InFusion (version 0.8), defuse (version 0.8.0), Chimpipe (version 0.9.8), PRADA (version 1.1), MapSplice (version 3.0), FusionCatcher (version 1.30), FusionHunter (version 1.4), EricScript (version 2.1), JAFFA-Assembly (version 2.0), Tophat-Fusion (version 2.0), ChimeraScan (version 0.4.5), SOAP-fuse (version 1.27), etc. Out of all these, STAR-Fusion and nFuse have higher true positive (TP) results [88]. To identify the fusion genes, all the above-mentioned tools can be used with their own pros and cons. Further, to screen the fusion mRNA, including the miRNA target sites, or to understand the mRNA:miRNA interactions, several other tools are used. These tools include miRanda, TargetScan etc. These are web-based tools and downloadable programs to predict miRNA based on the sequence given. The miRanda tool is a web-based tool where a user can provide an miRNA name and the symbol of the mRNA in a selected organism. The result shows the potential target mRNAs and alternative mRNA isoforms of the input miRNA. The miRanda tool effectively detects genes, including conserved or nonconserved sites [89,90,91]. The miRbase database is a web-based tool that has been used to detect miRNAs in fusion transcripts. Here, giving the mRNA fusion transcript sequence will help to identify the target miRNA sites in the fusion mRNA [40]. To study the functional effects of fusion genes, TargetScan can be employed. In breast tumors, miR-21-5p with 3′VMP1 fusion transcripts were predicted using TargetScan. It showed that the inclusion of the intronic miRNAs in host gene 3′ fusion transcripts can have functional effects through the deregulation of target genes [4].

## 8. Clinical Applications of Fusion Gene-miRNA Interplay

The miRNA profiling is useful in differentiating the tumor tissues from normal healthy tissues in rhabdomyosarcoma (RMS). MiRNA expression profiles are used to comprehend the difference between fusion-positive tumors and fusion-negative tumors in RMS. In rhabdomyosarcoma, miRNA expression profiling aids in studying the mRNA networks that are correlated with patient outcome profiles and fusion gene expression. In RMS, the fusion protein PAX3-FOXO1 impacts the miRNA expression of numerous miRNAs. miR-9-5p expression is affected by the fusion protein PAX3-FOXO1 in RMS. The miRNA expression profiles are useful in the subtyping of RMS. Moreover, they have identified new miRNAs that are independently associated with fusion-positive tumors and fusion-negative tumors. This study shows a possible interplay between fusion proteins and miRNAs in RMS. Understanding the interrelation between miRNA expression and fusion genes in fusion-driven tumors is useful in designing the therapeutics [92]. The identification of such cancer-specific driver fusion genes can become a future therapeutic target to treat cancer. Further studies are required to unveil the interplay between miRNAs and fusion genes, and understanding their mechanisms will further broaden our insights into various therapeutic applications [85].

## 9. Future Perspectives

Decrypting the multifaceted oncogenic pathways governed by fusion transcripts is imperative to comprehending their interactions with miRNAs. The investigation into whether fusion genes trigger aberrant miRNA expressions, constituting one of their critical oncogenic functions in driving carcinogenesis, necessitates thorough exploration. In order to evaluate the intricate interplay between fusion genes and miRNAs, it is essential to ascertain whether the fusion proteins can exert a direct or indirect influence on the dysregulation of miRNA networks. A second essential aspect involves probing whether the disruption of miRNA regulation on chimeric transcripts, resulting from fusion-induced inhibition of 3′UTR miRNA binding sites, contributes to unbridled tumorigenic signaling. Furthermore, a pivotal inquiry is whether fusion genes possess the capability to directly modulate oncomiRs and tumor suppressor miRNAs, thereby expediting the tumorigenesis. Ultimately, the assessment of fusion events leading to miRNA anomalies associated with cancer becomes paramount, irrespective of the protein-coding potential of the resulting fusion transcript. More research needs to be conducted in this subdomain of fusion genes affecting miRNA expression, which could be helpful in designing new therapies for cancer.

## Figures and Tables

**Table 1:** List of fusion genes regulating the aberrant expression of miRNAs and causing cancer.

S. No.	Fusion Gene	Dysregulated miRNA	Cancer Type	Reference
1	*BCP-ALL*	miR-125b-1	Myeloid cancer	[7]
2	* Translocation at 7q32.3	miR-29	ALK-negative large cell lymphoma	[8]
3	*PAX-FOXO1*	miR-29-a-3p	Alveolar rhabdomyosarcoma	[9]
4	* Deletion at 13q14	miR-15/16	Chronic lymphocytic leukemia	[10,11]
5	* t(8;17)(q24;q22)	miR-142	Acute promyelocytic leukemia	[12,13]
6	* Translocation at 12q15	Let-7 miRNA	Mesenchymal and benign tumors	[14,15,16]
7	*BCL6-LPP*	miR-28	Primary Central Nervous System Lymphoma	[17]
8	*BCL-ABL*	miR-17-92	Chronic myeloid leukemia	[14,18]
9	* Translocation at 17q23.2	miR-21	Glioblastoma	[14,19,20,21,22]
10	* t(8;14)(q24;q32)	miR-155	B-cell lymphoma	[23,24]
11	*AML1-ETO*	miR-223	Acute myeloid leukemia	[25]

(* Certain genetic rearrangements do not produce fusion genes but result in aberrant expression of miRNAs).
